# COVID-19 in a region of Cameroon hit by armed conflict

**DOI:** 10.11604/pamj.2022.41.32.32587

**Published:** 2022-01-12

**Authors:** Andreas Ateke Njoh, Eric Mboke, Shalom Tchokfe Ndoula, Hassan Ben Bachir, Raoul Nembot, Cornelius Chebo, Adidja Aman, Yauba Saidu

**Affiliations:** 1 Expanded Program on Immunization, Ministry of Public Health, Yaounde, Cameroon,; 2 Faculty of Science, University of Buea, Buea, Cameroon,; 3 Department of Family Health, Ministry of Public Health, Yaounde, Cameroon,; 4 Regional Delegation of Public Health for the Northwest Region, Bamenda, Cameroon,; 5 Faculty of Medicine and Biomedical Sciences, University of Yaounde I, Yaounde, Cameroon,; 6 Clinton Health Access Initiative, Yaounde, Cameroon,; 7 Institute for Global Health, University of Siena, Siena, Italy

**Keywords:** COVID-19, surge, immunization, armed conflict, Northwest Region, Cameroon

## Abstract

**Introduction:**

the emergence of more transmissible SARS-CoV-2 variants like Delta and Omicron have triggered the next wave of COVID-19 in many parts of the world. Here we report a surge in COVID-19 cases and deaths in the Northwest (NW) Region of Cameroon, which is plagued with low immunization coverage and armed conflict.

**Methods:**

a cross-sectional study was conducted in September 2021 and data on COVID-19 cases and vaccination were reviewed from the Ministry of Health database from January 1^st^, 2020 to September 4^th^, 2021. The security situation of the region was obtained from the districts and regional health managers. Data were analyzed with MS Excel and results presented as trends and proportions.

**Results:**

since the onset of COVID-19 pandemic, there is an increasing prevalence in cases in the NW. Between epidemiological week 34-35 of 2021, there was a surge in COVID-19 cases in the NW. More than 70% of all COVID-19 related deaths reported in the country during epidemiological week-35 were recorded in this region. Despite this high mortality, COVID-19 vaccine uptake remains very low in the region. Indeed, just 0.6% of the 962,036-target population 18-years and above are fully immunized after 6-months of vaccination.

**Conclusion:**

though the country´s epi-curve does not suggest a third wave currently, the NW is experiencing a steady COVID-19 case surge amid insecurity and the circulation of the Delta variant. There is therefore a need to adopt innovative strategies to improve immunization and strengthen other SARS-CoV-2 preventive measures in this region.

## Introduction

COVID-19 remains one of the greatest public health challenges in recent times. As of September 7^th^, 2021, the disease has been confirmed in over 222 million people, and has resulted into over 4.6 million deaths worldwide [1]. Since the emergence of more transmissible severe acute respiratory syndrome coronavirus 2 (SARS-CoV-2) variants, like the Delta and recently the Omicron first reported in South Africa on November 25^th^, 2021, the disease has continued to create panic in the world [2]. The emergence of the alpha, beta, and delta SARS-CoV-2 variants were associated with more infections and more countries experiencing their next waves of the disease characterized by an increased morbidity and mortality particularly with the Delta variant [3]. In Cameroon, the first case of COVID-19 was reported in March 2020 in the capital city, Yaoundé [4]. Since then, the number of cases has been increasing, and as of September 2021, the country has registered nearly 90,000 confirmed cases with 1,357 deaths [5]. Until now, most of the COVID-19 cases were limited to two of the most populous regions in the country- the center and littoral regions [5]. By August 13^th^, 2021, Cameroonian health authorities reported the circulation of the Delta variant in the country following its isolation from samples collected between May and July, 2021 in the Littoral and Center Regions [6,7]. Despite its isolation in these two regions, the variant is yet to be reported from other regions of Cameroon, including the northwest (NW). The NW Region has a total of 19 health districts and each of these districts is led by a district medical officer. Each health district is further sub-divided into health areas (247 health areas in total for the region), which are led by a chief of health area, who by default is the head of the leading health facility within the health area. Each of these health areas has at least one health facility which could be public or private [8]. Also, in each health area, there is at least one health facility that carries out immunization services.

With the emergence of COVID-19, care centers were set up for the management of cases [9] across the NW Region. In April, 2021, COVID-19 vaccination centers were set up from among the preexisting vaccinating sites to support the delivery of COVID-19 vaccines to persons 18 years and above according to the national guideline. In total, 23 vaccination centers were identified and accredited across the region, with each health district having at least one vaccination site. These sites began delivering Sinopharm and Covishield vaccines in April, 2021 and these vaccines were given in two doses separated by 3 weeks and 8 weeks respectively for full immunization. Johnson and Johnson vaccine was later added in July 2021, given as a single dose for full immunization. Vaccination services against COVID-19 are offered in these fixe vaccination sites on daily basis and in the community during outreach vaccination activities. In addition to the routine vaccination against COVID-19, the region has benefitted from two national vaccination campaigns that took place during epi week 18 and epi week 27. This region has conditions that may enhance the propagation of recent SARS-Cov-2 variants like Delta and Omicron. These include factors associated with the ongoing sociopolitical crisis for over 5 years now. This crisis has resulted into massive internal displacement of the population, looting and destruction of health facilities [10] and killing of healthcare workers, which collectively have led to a disruption of the healthcare system in the region [8,10]. Given the risk associated with the circulation of these new variants and particularly the Delta variant reported to be circulating in Cameroon for over 4 months now [6,7], we decided to assess the evolution of COVID-19 cases and deaths in the NW Region in a context of armed conflict and low COVID-19 vaccination coverage.

## Methods

**Study setting:** this study was carried out in the NW Region of Cameroon. This region is a home to close to 2.5 million inhabitants, majority of whom reside in rural areas where they are involved in farming for their livelihood. Over 40% of the population is aged 18 years and above. In this region, most functional health facilities can easily identify SARS-COV-2 through rapid diagnostic antigen tests.

**Study procedure:**the data for this cross-sectional study were collected for the period of January 1^st^, 2020 to September 4^th^, 2021. COVID-19 surveillance data were obtained from weekly epidemiological reports from the region and the national public health operation center. The epidemiological week also known as epi week was used to report the emergence of COVID-19 cases for 2020 and 2021 per week and it was also used to report the trend in COVID-19 vaccination coverage. Each epi week begins on a sunday and ends on the following saturday. So, the first epi week for 2020 started on sunday December 29^th^, 2019 and ended on saturday January 4^th^, 2020. The second epi week ran from Sunday January 5^th^ to Saturday January 11^th^, 2020. The rest of the epidemiological weeks followed the seven days series till the last epi week (53) of 2020 that started on Sunday December 27^th^, 2020 and ended on Saturday the January 2^nd^, 2021. So, Epi week-1 of 2021 ran from Sunday January 3^rd^to Saturday January 9^th^ January, 2021 and the rest of the epi weeks followed the same order.

COVID-19 vaccination data were obtained from the district health information Software (Dhis)-2. A pretested questionnaire was used to abstract key variables, including the security profile of the health districts of the region from 2020 to September, 2021. This security situation was updated to the already existing data for the previous four years as reported earlier [8]. This security classification considered the periodicity of arm violence, population displacement due to the armed conflict and health service accessibility challenges. Areas in low insecurity were districts in this region that were not experiencing armed conflict, population movement or health service accessibility challenges linked to the conflict. Areas in moderate insecurity are districts that experienced periodic arm violence with minimal population displacement and some health access challenges linked to the conflict within the year of interest. Meanwhile areas in high insecurity are those that experienced recurrent arm violence with mass population movement, looting of health facilities and health service accessibility challenges linked to the violence. The questionnaires were filled by the regional and district managers of the NW Region. The investigation team then resolved any identified data discrepancy by directly calling the regional head or the district medical officers concerned.

**Data management and analysis:** the data were analyzed with Microsoft Office Excel 2019 and summary statistics was used to estimate incidence, prevalence, case fatality and vaccination coverage. This was a secondary study that used data abstracted from the existing data of the Ministry of Health. So, no ethics approval and consent to participate were required.

## Results

**Evolution of security profile:** the region began experiencing insecurity in late 2016 following the onset of sociopolitical crisis that degenerated into an armed conflict [8]. Unlike in 2019 when we had two districts (Bamenda and Tubah) that were in moderate insecurity as reported earlier [8], we observed that in 2020 and 2021, all districts of the region are in high insecurity.

**Figure 1:**
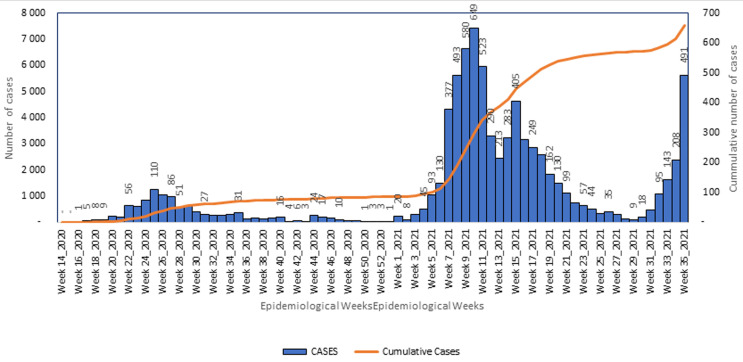
trend in the evolution of COVID-19 cases in the Northwest Region, Cameroon, 2020-2021

**Figure 2:**
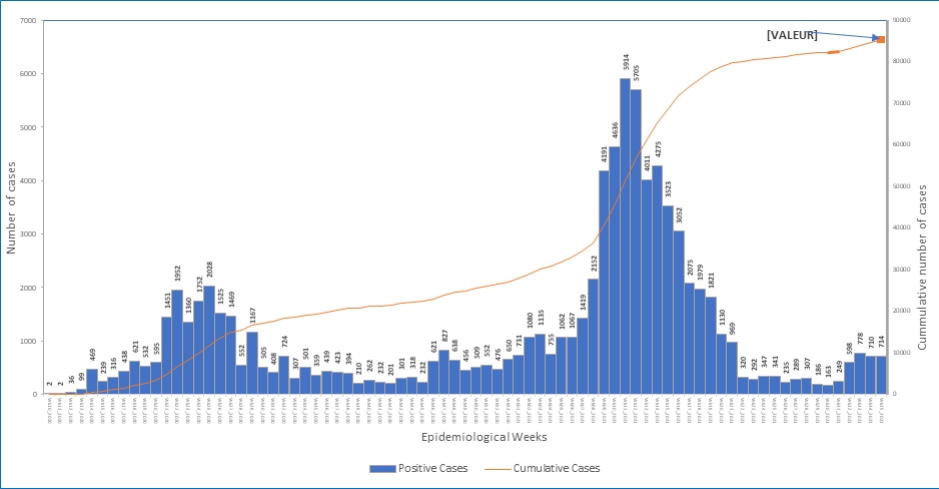
trend in the evolution of COVID-19 cases in Cameroon, 2020-2021

**Evolution of COVID-19 cases in the region:** by September 4^th^, 2021 (end of epidemiological week 35), the region had registered a total of 7,524 confirmed cases of COVID-19 (Figure 1), 53% of whom are males. Of this number 84% have recovered. As illustrated in Figure 1, the region experienced a surge in its epi-curve, marked by a sudden rise in the number of new cases in 2021, from just nine in epidemiological week 29 to 491 in epidemiological week 35. Also, Bamenda health district recorded the highest incidence in the region at week 35 this year and the highest prevalence (Table 1). Over 69% of cases reported in Cameroon on epi week-35, 2021 (Figure 2) originated from the NW Region. This surge in COVID-19 cases in the NW, however, does not seem to be reflected in the national epi curve, as the curve shows a minimal increase in new cases during this period (Figure 2).

**Table 1:** COVID-19 cases per health district of the Northwest Region

Health districts	Total population	Incidence/1000 Epi week 35	Prevalence/1000	Case fatality (%)
AKO	58862	0,0	0,8	0,0
Bafut	60843	0,5	2,5	0,6
Bali	37103	0,1	5,0	0,5
Bamenda	429419	0,6	7,0	5,8
Batibo	95150	0,0	0,4	2,4
Benakamu	60794	0,0	0,6	0,0
Fundong	158243	0,1	5,0	6,0
Kumbo East	159554	0,4	2,9	7,8
Kumbo West	113508	0,1	5,3	2,6
Mbengwi	56469	0,0	2,5	2.8
NDOP	275603	0,0	1,3	1,1
Ndu	95914	0,3	2,2	2.4
Njikwa	23017	0,0	0.8	0.0
Nkambe	147968	0.0	3,3	4,7
Nwa	65599	0,0	0,9	0,0
Oku	101536	0,0	1,2	2,5
Santa	101176	0,2	2,5	1,2
Tubah	69999	0,2	5,6	0,8
Wum	135546	0,1	1,9	5,8
North West	2246303	0,2	3,4	4,4

**Figure 3:**
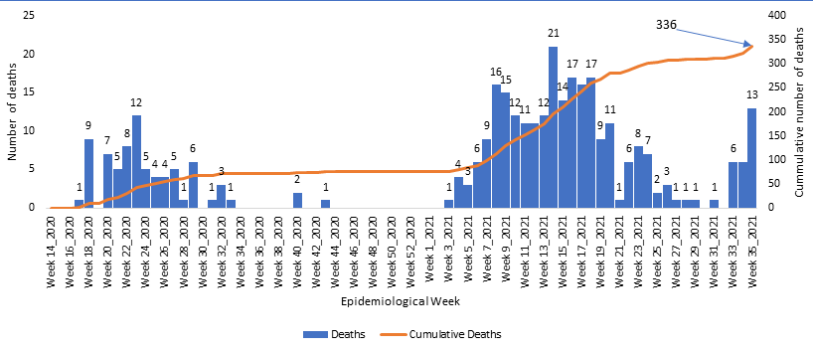
trend in the evolution of COVID-19 related deaths in the Northwest Region, Cameroon, 2020-2021

**Evolution of COVID related deaths in the region:** overall, the region has recorded 336 COVID-19 related deaths. Figure 3 illustrates the evolution of deaths during the first and second waves in the region. It also depicts in 2021, a jump in the number of deaths between week-29 and week-35, with the number rising from just one in week-29 to 13 in week-35. Over 50% of the deaths during week-35, 2021 in the NW were recorded in the regional capital of Bamenda. The region further accounted for over 65% of all COVID-19 related deaths in Cameroon during the week. Overall, NW currently has a case fatality rate of 4.4% (Table 1), which is two times the national average.

**Figure 4:**
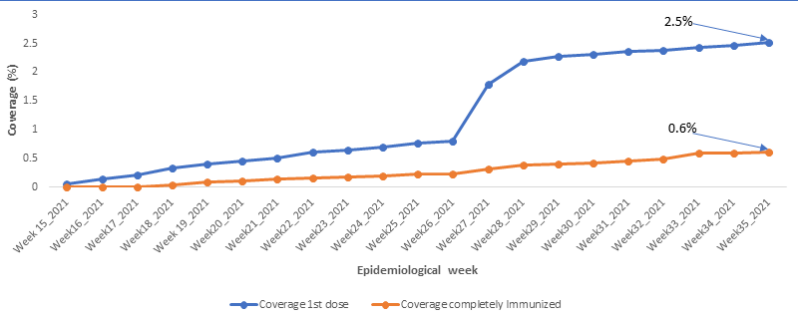
evolution of COVID-19 vaccination coverage for persons aged 18 years and above

**COVID-19 vaccines uptake in the region:** the COVID-19 immunization coverage for the NW remains very low with just 2.5% of the target population (persons aged 18 years and above: 962 036) receiving at least a dose of the available COVID-19 vaccine by epi week 35, 2021. Also, just 0.6% of this target population has been fully immunized by the end of epidemiological week-35 of 2021 as presented in Figure 4.

## Discussion

The NW Region of Cameroon, like the rest of the country, was affected by the COVID-19 pandemic in early 2020. Since then, the region has experienced two waves of the pandemic [5] with the last case surge recorded in the first half of 2021 (Figure 1). The region is recently experiencing a surge in its epi-curve, with the number of new cases doubling on a weekly basis since week-30 of 2021 (Figure 1). In week-34 and 35, more than 60% of all COVID-19 related deaths recorded in the country were reported from this region [5]. The highest proportion of new cases and deaths reported in the region, were recorded in Bamenda health district which is the economic and administrative headquarter and has the main referral hospital of the region. It is worth noting that the sudden rise in cases and deaths is occurring after the detection of the Delta variant in the country and this raises the question on whether the more infectious Delta variant with a higher risk of severe disease, hospitalization, and death is not already circulating in the region [11,12]. The situation in this region remains alarming for the country, whose national epi curve does not suggest a third wave of COVID-19 currently (Figure 2). Laboratory capacity to investigate this surge are lacking and at this point, it may be difficult to rapidly detect the circulation of new SARS-COV-2 variants in this region. Though from June, 2020, like the rest of the country, the NW benefitted from rapid antigen screening test for SARS-COV-2 that has permitted quick identification of cases in all major health facilities across the region [9], the region cannot yet identify the particular variant responsible for infections. To detect the variant, samples collected from suspected cases must be sent out of the region for sequencing, and this may take long for the results to be made available. Indeed, when the Delta variant was first suspected in the country, samples were collected in May, 2021, but the confirmation on the circulation of this variant was only published three months later [6,7]. This long turnaround time coupled with the prevailing insecurity in the region [8] may result into more infections and deaths from this varus.

The current case surge can also be partly linked to the massive internal displacement of the population [8,10] over the years due to the worsening security context, that exposes the population to a higher risk of infectious diseases. Following mass population movement, asymptomatic or pre-symptomatic persons, who are often missed and not isolated, can spread the virus to more persons as they move from one location to another [13] in search of refuge. Furthermore, with the distortion in the health system partly caused by looting of health infrastructures, killing of health staff and closure of some health facilities [8,10], some persons with milder forms of the disease turn to remain within their safe zones with loved ones and this can favor the spread of SARS-COV-2. Also, with the conflict, the region experiences periodic movement of the population from sites experiencing fierce conflict to safer parts of the country and this population subsequently returns when there is relative peace. These movements can favor the transportation of the virus [13,14] into the region. Despite this concerning situation, vaccine uptake remains low in the region. Currently, just 2.5% of the population aged 18 years and above in the region have received at least one dose of any of the available COVID-19 vaccines. In addition, just over 0.6% have received the full doses recommended to ensure full immunization for either of the available vaccines. This limited vaccine uptake continues to flourish in this setting, partly due to vaccine hesitance [15] and due to challenges related to delivering vaccines in this security compromised area [8]. As a result, the risk of infection and severe disease in this population remains particularly high considering the evidence of the circulation of the Delta variant in the country [16]. To counter this risk, there is the urgent need to scale up COVID-19 vaccination in this region to prevent severe disease and limit the emergence of new variants [17,18]. There is therefore a need to use innovative approaches adapted to the local context to help reach the vaccination target. The use of community participation at every level of the vaccine rollout, for instance, during planning, generation of resources, vaccination and monitoring and evaluation can help strengthen service delivery and improve vaccination coverage [19,20] against COVID-19.

Given the recurrent population displacement experienced in this setting, carrying out vaccination at transit points such as bus stations and refuge sites can help improve vaccine uptake [19]. Also, ensuring that vaccination teams are made up of persons among the internally displaced can help locate the target population and improve vaccine acceptance. In addition, carrying out supplementary immunizations and periodic intensification of routine immunization activities during periods of relative tranquility might further help improve vaccine uptake [21,22] and reduce the burden of COVID-19 on this population. To the best of our knowledge, this is the first systematic report on the surge of COVID-19 cases in a region that has been suffering from an armed conflict for over 5 years [8]. Despite this contribution, this piece of work has some limitations. Firstly, the primary data were collected in a context of insecurity, and this could have introduced some bias into the data. Also, the denominators used in calculations were based on estimates from the central level, which may not necessarily reflect the real situation on the ground bearing in mind the current population movement reported in this region since the onset of the sociopolitical crisis [10,23]. Despite these limitations, we remain hopeful that this study will stimulate more research to clearly elucidate the reasons for the surge in COVID-19 cases in the NW. This may be important now that there is evidence of the Delta variant circulating in the two major cities in Cameroon associated with the risk of importation of other variants like the Omicron. Further research may also help elucidate why the national epi curve does not suggest a third wave, even though there is evidence of a third wave in the NW at this time.

## Conclusion

The findings in this study suggest a sudden COVID-19 case surge and deaths in the NW Region of Cameroon in the context of armed conflict, circulating COVID-19 Delta variant and low vaccination coverage. Though the Delta variant has not yet been isolated in this region, the risk of a local outbreak linked to new SARS-CoV-2 variants remains. There is, therefore need to strengthen SARS-CoV-2 genomic surveillance in this region and to develop strategies to improve COVID-19 immunization coverage and the respect of other measures to limit the spread of the disease.

### What is known about this topic

- *With the emergence of new SARS-CoV-2 variants like the Delta variant and the Omicron variant, many more persons around the world are being infected and more countries are experiencing their next waves of the disease;*
- *These new waves are characterized by an increased morbidity and mortality particularly with the Delta variant;*
- *However, some countries like Cameroon appear to present a relatively few new cases of COVID-19 and the national epi-curve does not yet suggest the occurrence of a third wave by epi week-35, 2021*.

### What this study adds

- *Our findings suggest that though the country's epi-curve is not suggestive of a third wave of COVID-19 at this time, the NW Region which is hit by armed conflict for a couple of years now is greatly affected by COVID-19;*
- *The region experienced a jump in the number of deaths between week-29 and week-35, from just one to 13 and accounted for over 65% of all COVID-19 related deaths in Cameroon during week-35;*
- *So, this region likely requires special interest to mitigate the effect of COVID-19 in the context of the current prevailing armed conflict*.
